# Pediatric Ulcerative Colitis in Siblings

**DOI:** 10.7759/cureus.40829

**Published:** 2023-06-22

**Authors:** Divya Mamootil

**Affiliations:** 1 Internal Medicine, Ascension St. Agnes Hospital, Baltimore, USA

**Keywords:** biologic therapies, steroid treatment, immunosuppression therapy, pediatric genetics, irritable bowel disease, ulcerative colitis (uc)

## Abstract

This case report discusses a nine-year-old female that presented with abdominal pain, diarrhea, and weight loss, suggestive of inflammatory bowel disease (IBD). She had an older brother previously diagnosed with ulcerative colitis (UC), which raised suspicion that she may have the same condition. CT scan of the abdomen/pelvis showed signs of bowel thickening. Stool studies revealed elevated inflammatory markers including lactoferrin and calprotectin, as well as occult blood. She underwent a colonoscopy and rectal biopsy which further confirmed the diagnosis of ulcerative colitis. This article aims to discuss the clinical presentation, role of genetic factors, diagnostic workup, and therapeutic management of ulcerative colitis in the pediatric population.

## Introduction

Ulcerative colitis (UC) is a chronic and idiopathic inflammatory bowel disease (IBD) that causes ulcers and inflammation in the colon. The classic triad of IBD is abdominal pain, diarrhea, and weight loss, with the addition of rectal bleeding in UC patients. The pediatric population makes up 15-20% of all IBD cases in North America [1], and the incidence has continued to increase over the years. Pediatric IBD is classified based on the age they are diagnosed; very early onset (VEO) is < 10 years old and early onset (EO) is 10 to < 17 years old [2]. VEO cases tend to have increased severity of the disease or more extensive involvement in the colon, while EO cases present similarly to adult IBD [2]. Adult UC is mainly confined to the rectum or left side of the colon, while children generally present with pancolitis [3]. Pediatric IBD is suspected with persistent (≥4 weeks) or recurrent (≥2 episodes in 6 months) of these symptoms [3]. Children mainly complain of abdominal pain at the start of their diagnosis. 10-40% of pediatric patients can initially present with non-specific symptoms such as growth failure [3].

## Case presentation

A nine-year-old previously healthy Caucasian female presented with a chief complaint of intermittent episodes of diarrhea and abdominal pain for three months. She also noticed bright red blood and mucus in her stools a few days prior to hospital admission. Associated symptoms included fatigue, generalized malaise, difficulty sleeping, and lack of appetite during that week. She denied any fever, chills, nausea, vomiting, recent travel, or consumption of poorly cooked food. Her past medical history only consisted of occasional upper respiratory symptoms and otitis media. She did not have allergies and was not taking any medications. Her family history was significant for a 13-year-old brother that had similar complaints of bloody stools a year ago and was diagnosed with ulcerative colitis at age 12. 

Upon admission, she was afebrile with a blood pressure of 100/60 mmHg, a pulse of 90 beats/minute, a respiratory rate of 12 breaths/minute, and pulse oximetry of 100% on room air. Her BMI was 15.42. On the physical exam, there were no signs of scleral icterus, jaundice, pallor, rashes hepatomegaly, or splenomegaly. The cardiopulmonary exam was within normal limits. Her abdomen was non-distended, and soft, with mild generalized tenderness on palpation, without rigidity or guarding. Murphy's sign was negative.

Laboratory findings were significant for white blood cell (WBC) of 12.2 K/uL (reference range: 4-11 K/uL), hemoglobin 9.2 g/dL (reference range: 12-15 g/dL), lactic acid of 1.9 mmol/L (reference range: 0.5-1.6 mmol/L), Thyroid stimulating hormone (TSH) 3.11 mIU/L (reference range: 0.27-4.2 mIU/L), and free thyroxine (T4) of 1.2 ng/dL (reference range: 0.93-1.7 ng/dL). Aspartate aminotransferase (AST) was 38 U/L ( reference range: 0-34 U/L) and Alanine aminotransferase (ALT) was 24 U/L (reference range: 0-36 U/L). Inflammatory markers were elevated including Erythrocyte sedimentation rate (ESR) of 34 mm/hour (reference range: 0-20 mm/hour) and c-reactive protein (CRP) of 15.2 mg/L (reference range: 0-5 mg/L) (Table 1).

**Table 1:** Inflammatory markers (blood test)

Inflammatory markers (blood test)	Value (reference range)
Erythrocyte sedimentation rate (ESR)	34 mm/hour (0-20 mm/hour)
C-reactive protein (CRP)	15.2 mg/L (0-5 mg/L)

Stool samples were taken and revealed that the fecal lactoferrin assay was positive. Fecal calprotectin was 514 mcg/g (reference range: 0-50 mcg/g). Stool WBC showed 18-20 white blood cells/HPF. Occult blood was also positive. Ova and parasites were negative. Clostridium difficile toxin and antigen were both negative (Table 2).

**Table 2:** Stool studies

Stool studies	Value (reference range)
Lactoferrin assay	positive
Calprotectin	514 mcg/g (reference range: 0-50 mcg/g)
White blood cells (WBC)	18-20 white blood cells/HPF
Occult blood	positive
Ova and parasite	negative
Clostridium difficile toxin and antigen	negative

**Figure 1:**
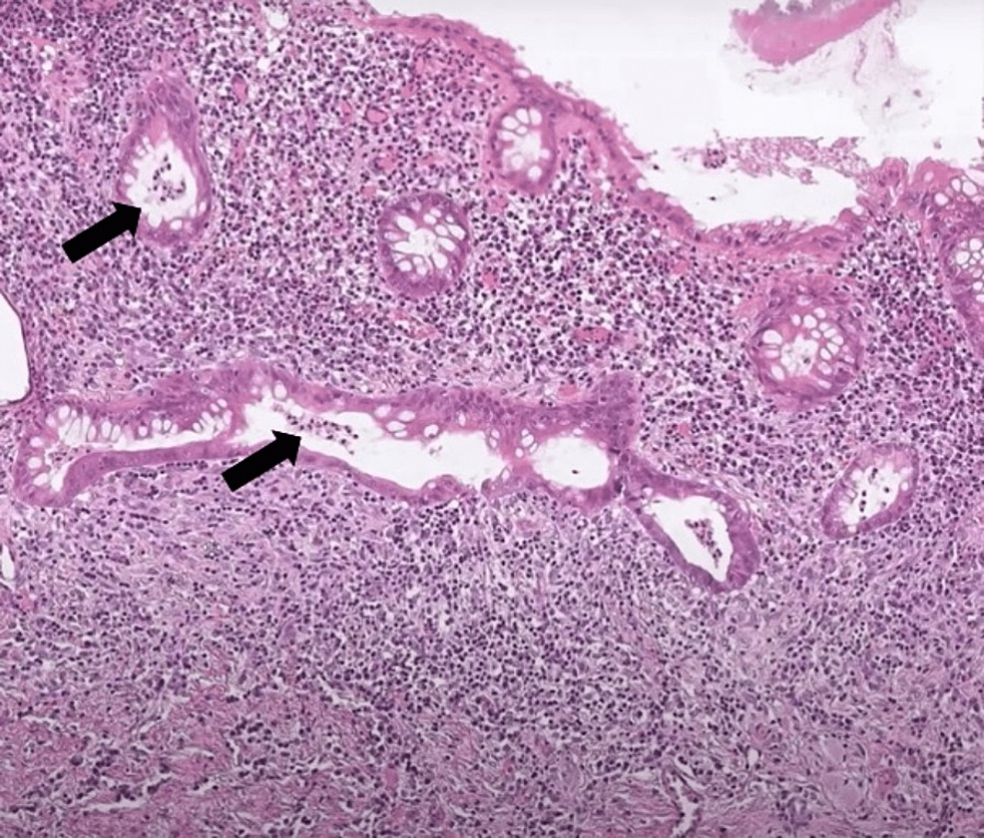
Hematoxylin and eosin (H&E) histology stain (100x magnification) of rectal biopsy showing crypt abscesses with varying morphologies, atrophy, and neutrophil infiltration

CT scan of the abdomen/pelvis showed bowel wall thickening of the descending colon and rectum to 5.2 mm with irregularity of the wall contour and luminal narrowing. She underwent a colonoscopy, which revealed inflammation and friable mucosa with vascular effacement in the rectum and descending colon. Rectal biopsy was done and the pathology report showed crypt abscesses of varying sizes, crypt atrophy, and infiltration of polymorphonuclear leukocytes (<50% invasion), consistent with a diagnosis of ulcerative colitis (Figure 1).

Based on her clinical symptoms and pathology findings, her pediatric ulcerative colitis activity index (PUCAI) was classified as a score of 45 which is moderate disease severity. She was started on Prednisone 1 mg/kg daily concomitant with Mesalamine 50 mg/kg daily for one month with a gradual steroid taper. She was able to tolerate this well with improvement in her symptoms.

## Discussion

Gene studies

There is an estimated 7.9-12% prevalence of IBD among family members [4]. There are many studies that show strong gene linkage in patients with UC or Crohn’s, and a high monozygotic twin concordance rate of 15.4% compared to 3.9% dizygotic [4]. However, it is still a polygenic disease affected by environmental factors. One study showed siblings can influence the transmission and severity of clinical presentation of UC and Crohn’s disease [5]. They looked at the role of environmental exposures in relation to increased risk or protection from developing IBD within a specific window of susceptibility. Findings were remarkable for older siblings having a higher risk of developing UC [5]. One reason for this is that older siblings tend to develop asthma, allergies, and other atopic diseases more often due to the first exposure to certain antigens and environmental factors compared to their siblings. When younger siblings come along, they are in turn exposed to the same antigens and also have a high risk of developing the same disease, which may have more severe manifestations [5]. 

Genetics plays a strong role in the predisposition of some pediatric patients to developing UC. Genome-wide association studies (GWAS) in the past have shown a strong association of specific MHC class II alleles with UC, such as HLA DRB1*01:03 and HLA DQA1 [6]. These studies showed less heterozygosity in HLA genes, suggesting it would be protective for patients to have broader antigen recognition. This is particularly important for the pediatric population, as they are more prone to developing extensive colonic involvement, especially under the age of eight [7]. Another important factor to consider is the integrity of the intestinal epithelial barrier to prevent the development of colitis. Studies have shown patients with UC to have a deficiency in the MUC2 gene, which is responsible for producing mucins that help protect the lining of the colon [8]. UC patients have also had polymorphisms in ECM1, which is another gene that controls the strength of the epithelial basement membrane in the colon [9]. IBD patients tend to have increased inflammatory markers that cause colitis, such as interleukins that create an autoimmune response to attack the colon when they perceive a threat. IL-6, IL-12, and IL-23 exacerbate inflammation of the colon in IBD patients [10]. 

Diagnostic workup

A complete blood count (CBC) should be done in all patients to evaluate anemia, leukocytosis, or thrombocytosis. One study showed the combination of anemia and thrombocytosis in pediatric patients with IBD had a positive predictive value of 90% and a negative predictive value of 81%. [11]. ESR and CRP should also be tested as they are inflammatory markers, although they can also be elevated in infectious colitis as well. Stool samples are a common diagnostic tool used in the workup of IBD, as they can identify specific inflammatory markers. Lactoferrin is a glycoprotein that binds to and transports iron and is also a modulator of the immune system. There are elevated levels of lactoferrin when there is intestinal inflammation in IBD. One study indicated elevated lactoferrin had a sensitivity of 78% and specificity of 90% in IBD patients [12]. Osteoprotegerin is responsible for preventing osteoclast formation by blocking the (receptor activator of nuclear factor kappa beta) RANK ligand, as well as playing a role in the apoptosis pathway. Its pro-inflammatory effects identify colon inflammation in IBD patients. A study in children with IBD showed fecal osteoprotegerin had a sensitivity of 71% and specificity of 69% [13]. Calprotectin and S100A12 are other proinflammatory markers from a group called DAMP (damage-associated molecular pattern molecules) [14]. These markers are overexpressed when there is inflammation in the colon. A study revealed fecal calprotectin is significantly elevated in pediatric IBD patients with a sensitivity of 100% and specificity of 67% [15]. With all cases of bloody diarrhea, infectious causes must be ruled out first. Colonoscopy and upper endoscopy are essential to differentiate between Ulcerative Colitis and Crohn’s disease. Visualization of the ileum is also necessary to rule out pancolitis [3].

Therapeutic management

The PUCAI is a tool used to predict how children will respond to therapy based on disease severity. The mild disease has a score between 10 and 30. Moderate disease has a score between 35 and 60. Severe disease has a score greater than 65. Severe disease is characterized by ≥6 bloody stools/day and one of the following: anemia, fever, tachycardia, or elevated ESR [16]. There are several different treatment modalities used for UC usually based on their disease severity. Pediatric patients with mild disease usually begin treatment with an oral 5-Aminosalicylic acid (5-ASA) preparation such as mesalamine as first-line therapy to induce remission. Rectal mesalamine can also be used in conjunction with oral formulation to improve clinical outcomes [1]. One thing to be aware of is the possible transient exacerbation of diarrhea in the first few weeks following treatment, as this is a common side effect [1]. Oral glucocorticoids such as prednisone are often used as a second-line treatment for mild to moderate UC when unresponsive to mesalamine, or first-line therapy for moderate disease [17]. Intravenous (IV) glucocorticoids may also be used if unresponsive to oral therapy. Thiopurine therapy such as Azathioprine or 6-mercaptopurine may be added to maintain remission in patients that are dependent on steroids. [17].

Patients with severe UC with PUCAI score >65 are preferentially treated with IV steroids [16]. The scores are reassessed every two days to determine if additional therapy is warranted. For example on day five, if the patient is still unresponsive with a score >65, infliximab or IV cyclosporine may be given as second-line therapy [16]. If the score has improved to <35 on day five the patient may be switched to oral steroids [17]. Vedolizumab is a second-line biologic therapy that targets inflammation by blocking the recruitment of lymphocytes specific to the gut [18]. There is some evidence that a combination of broad-spectrum antibiotics (metronidazole, amoxicillin, doxycycline, and vancomycin [MADoV]) has been successful in children with UC that have failed other therapies [19]. There have also been a few studies in children with fecal microbial transplantation (FMT), as a proposed treatment of UC [20]. Pediatric patients with the refractory disease will have PUCAI scores that remain above 65 despite other therapies, so they may require surgical referral for a subtotal colectomy and ileostomy [16]. Other indications for colectomy include complications such as toxic megacolon, perforation, and hemorrhage. 

## Conclusions

As the incidence of ulcerative colitis increases in the pediatric population, further studies will need to be done to investigate the role of genetics and environmental factors in their susceptibility to the disease and response to management. It is also essential to spread awareness about the disease and educate children and their families about how Ulcerative Colitis will affect their lives. With advances in technology, there is great hope for the future of pediatric patients. 
